# Rare Cancer-Associated Mutations May Affect the AF1 Phosphoregulatory Domain of RARγ

**DOI:** 10.3390/genes17091119

**Published:** 2026-09-15

**Authors:** Eliezer Kopf

**Affiliations:** Safety Unit, Weizmann Institute of Science, P.O. Box 26, Rehovot 7610001, Israel; eliezer.kopf@weizmann.ac.il; Tel.: +972-523046046

**Keywords:** RARγ, RARG, AF1 domain, phosphorylation, proteasomal degradation, somatic mutation, cancer genomics, epithelial malignancies, nuclear receptors, COSMIC

## Abstract

Background/Objectives: Retinoic acid receptor γ (RARγ), encoded by RARG, is increasingly recognized as a cancer-associated regulator through aberrant expression in multiple solid tumors and recurrent gene rearrangements in acute myeloid leukemia. While the oncogenic relevance of RARγ is well established, the potential involvement of its N-terminal activation function-1 (AF1) domain in human cancer remains poorly characterized. Previous biochemical studies demonstrated that phosphorylation of Ser66 and Ser68 in RARγ2, corresponding to Ser77 and Ser79 in canonical RARγ1, is required for ligand-dependent receptor activation, ubiquitination, and proteasome-mediated degradation. Methods: To determine whether this experimentally validated phosphoregulatory region is altered in human cancer, we interrogated the Catalogue of Somatic Mutations in Cancer (COSMIC v104, GRCh38) at single-residue resolution. Mutation data were integrated with clinical and pathological information obtained from the corresponding primary publications and public annotation resources. Results: Rare somatic mutations affecting the AF1 phosphoregulatory region were identified, including the missense variants p.S77L and p.S79L, the nonsense variant p.S79*, and the in-frame insertion p.S77_P78insLQ. These alterations occurred in independent epithelial malignancies, including cervical neuroendocrine carcinoma, head and neck squamous cell carcinoma, lung adenocarcinoma, bladder carcinoma, and gastric neuroendocrine carcinoma. Notably, the truncating p.S79* mutation was detected in five spatially distinct regions of a single EGFR-mutant lung adenocarcinoma, consistent with an early clonal event during tumor evolution. Rare cancer-associated mutations affect a previously characterized AF1 phosphoregulatory module of RARγ. These findings extend established RARγ regulatory biology into the context of human malignancy and broaden the spectrum of reported cancer-associated RARG alterations. While the functional consequences of these rare variants remain unknown, their occurrence within residues known to regulate receptor activation and turnover highlights the AF1 phosphoregulatory region as a candidate for future mechanistic investigation.

## 1. Introduction

Retinoic acid receptor γ (RARγ), encoded by RARG, is a member of the nuclear receptor superfamily that regulates cellular differentiation, stem-cell maintenance, proliferation, and tissue homeostasis through ligand-dependent transcriptional programs. Increasing evidence indicates that RARγ also contributes to tumorigenesis. Elevated RARγ expression has been reported in multiple malignancies, including prostate, hepatocellular, pancreatic, ovarian, renal, cholangiocarcinoma, and head-and-neck cancers, where increased receptor activity has been associated with cancer-cell proliferation, stem-cell maintenance, aggressive disease behavior, and adverse clinical outcome [1,2,3] (Table 1). In addition, recurrent RARG rearrangements define a distinct subset of acute myeloid leukemia characterized by impaired differentiation, resistance to retinoid-based therapies, and poor prognosis [4,5] (Table 1). Together, these observations establish RARγ as a cancer-associated gene and highlight its importance in both epithelial and hematological malignancies.

RARγ contains several functional domains, including an N-terminal activation function-1 (AF1) domain, a central DNA-binding domain (DBD), a hinge region, and a C-terminal ligand-binding domain (LBD). Most studies investigating the role of RARγ in human cancer have focused on receptor overexpression, altered signaling pathways, or oncogenic fusion proteins involving the DNA-binding and ligand-binding regions. Consequently, comparatively little attention has been directed toward the AF1 domain despite its established role in post-translational regulation and receptor turnover.

More than two decades ago, biochemical and molecular studies demonstrated that phosphorylation of Ser66 and Ser68 in RARγ2, corresponding to Ser77 and Ser79 in canonical RARγ1, is required for ligand-dependent receptor activation, transcriptional competence, ubiquitination, and proteasome-mediated degradation [6,7,8,9,10]. These residues form a functionally validated phosphoregulatory module within the AF1 domain that integrates TFIIH/CDK7-dependent phosphorylation, p38MAPK signaling, PI3K/Akt regulation, and proteasome recruitment. Experimental disruption of phosphorylation at these sites impairs receptor turnover and transcriptional activation, establishing this short AF1 segment as a critical regulatory element in RARγ biology [6,7,8,9]. The phosphoregulatory serines are embedded within a proline-rich sequence that may contribute to local structural organization and regulatory function.

Despite the well-established biochemical importance of this AF1 phosphoregulatory module, its involvement in human cancer has not been systematically investigated. While recurrent alterations affecting other regions of RARG, including gene rearrangements and expression changes, have been extensively documented [1,2,3,4,11,12], the occurrence of somatic mutations affecting the experimentally validated AF1 phosphoregulatory region remains largely unexplored.

Since that phosphorylation of Ser77 and Ser79 is required for normal receptor activation, transcriptional competence, and proteasome-mediated turnover [6,7,8], genetic alterations affecting these residues could potentially disrupt key regulatory mechanisms that maintain RARγ function. Such disruption may alter receptor signaling, protein stability, or cellular differentiation pathways relevant to malignant transformation and tumor progression [1,2]. Determining whether cancer-associated variants occur within this functionally validated regulatory module is therefore important for understanding whether direct genetic alteration of AF1 represents an additional mechanism of RARγ dysregulation in human cancer (Table 1).

In the present study, we interrogated the Catalogue of Somatic Mutations in Cancer (COSMIC v104, GRCh38) to determine whether rare cancer-associated mutations affect the AF1 phosphoregulatory domain of RARγ.

We identified somatic alterations involving the experimentally validated phosphoregulatory residues Ser77 and Ser79, including missense, nonsense, and in-frame insertion variants occurring in multiple epithelial malignancies. These findings extend observations from earlier experimental studies to human cancer genomics and demonstrate that rare cancer-associated mutations can occur within the AF1 phosphoregulatory domain of RARγ, a regulatory region that has received limited attention in cancer studies.

**Table 1:** Published evidence linking RARG alterations and functional domains to human malignancies.

Alteration	Domain/ Region	Malignancy	Major Finding	Reference
RARG overexpression	Whole protein	Prostate cancer and other solid tumors	Promotes proliferation and cancer-cell survival	[1]
RARG overexpression	Whole protein	Hepatocellular, cholangiocarcinoma, ovarian, pancreatic, renal and head-and-neck cancers	Associated with tumor progression and poor prognosis	[2]
RARG dysregulation	Whole protein	Multiple solid tumors	Supports stem-cell maintenance and oncogenic signaling	[3]
CPSF6-RARG, PML-RARG, NPM1-RARG and related fusions	DBD/LBD	AML	Distinct leukemia subtype associated with poor prognosis	[4]
NUP98-RARG fusion	DBD/LBD-containing fusion	AML	APL-like leukemia resistant to retinoid therapy	[11]
Multiple RARG fusion proteins	DBD/LBD	AML	Mechanistic role in leukemogenesis	[12]
Rare AF1 phosphoregulatory-region mutations (S77L, S79L, S79*, S77_P78insLQ)	AF1 domain	Epithelial malignancies	First report describing rare cancer-associated variants affecting the AF1 phosphoregulatory region of RARγ.	Kopf E. 2026; present manuscript (finding originated from analysis of publicly available COSMIC data conducted in the present manuscript rather than from an original patient cohort).

## 2. Methods

### 2.1. Study Design

The present work should be viewed as a descriptive cancer-genomic study that reports the occurrence of rare AF1-domain mutations rather than demonstrating their biological or oncogenic consequences.

### 2.2. Data Sources

Somatic mutations affecting RARG were retrieved from the Catalogue of Somatic Mutations in Cancer (COSMIC, version 104, GRCh38 assembly (15). Analysis focused on mutations affecting the experimentally validated phosphoregulatory region of the RARγ activation function-1 (AF1) domain, specifically residues Ser77 and Ser79 of canonical RARγ1. Clinical and pathological information associated with individual mutation records was obtained from the datasets referenced within COSMIC.

### 2.3. Isoform Mapping

Residue numbering was reported according to canonical human RARγ1 (NP_001036193.1). Because the major phosphorylation studies were performed using RARγ2, residue correspondence between isoforms was established using published sequence alignments. Ser66 and Ser68 of RARγ2 correspond to Ser77 and Ser79 of canonical RARγ1, respectively.

### 2.4. Mutation Identification and Annotation

All COSMIC entries affecting residues Ser77, Ser79, or immediately adjacent amino acids within the AF1 phosphoregulatory segment were manually reviewed. Mutation type, coding consequence, amino acid change, COSMIC identifier, tumor type, and available clinical annotation were recorded. Identified variants were classified as missense, nonsense, synonymous, or in-frame insertion according to the COSMIC annotation.

### 2.5. Clinical Annotation

Clinical and pathological characteristics of tumors harboring AF1-domain mutations were extracted from the corresponding source publications linked to COSMIC entries. Information reviewed included tumor type, histological classification, clinical stage when available, demographic characteristics, and relevant molecular features reported by the original investigators.

### 2.6. Post-Translational Modification Resources

The iPTMnet database was consulted to review experimentally validated post-translational modifications affecting the AF1 phosphoregulatory region and to identify previously reported variants involving the same residues. Information from iPTMnet was used solely for annotation of the phosphoregulatory context of the identified mutations.

### 2.7. Data Availability

All data analyzed in the present study were obtained from publicly available resources, including COSMIC (v104, GRCh38), iPTMnet, and the cited primary publications. No new sequencing data was generated during this study.

## 3. Results

The AF1 domain of RARγ contains a functionally validated phosphoregulatory segment centered on Ser77 and Ser79 of canonical RARγ1, corresponding to Ser66 and Ser68 of RARγ2. To determine whether this regulatory region is altered in human malignancies, COSMIC v104 (GRCh38) was interrogated for somatic mutations affecting the AF1 domain.

This analysis identified protein-altering mutations directly involving the phosphoregulatory residues, including the missense substitutions p.S77L and p.S79L, the truncating mutation p.S79*, and an in-frame insertion p.S77_P78insLQ (Table 2 and Table 3). These mutations were identified in independent epithelial malignancies, including cervical neuroendocrine carcinoma, head and neck squamous cell carcinoma, lung adenocarcinoma, bladder carcinoma, and gastric neuroendocrine carcinoma (Table 4).

The identified mutations affect residues previously shown experimentally to regulate ligand-dependent activation, ubiquitination, and proteasome-mediated degradation of RARγ, extending the relevance of this phosphoregulatory region from mechanistic receptor biology to human cancer.

### 3.1. Clinical Characteristics of Tumors Harboring AF1 Mutations

Clinical annotations of the affected tumors are summarized in Table 4. The identified mutations were detected across distinct epithelial malignancies and clinical contexts, suggesting that alteration of the AF1 phosphoregulatory region is not restricted to a single tumor lineage.

Particularly notable was the p.S79* truncating mutation identified in an EGFR-mutant lung adenocarcinoma. This mutation was detected in five spatially distinct tumor regions from the same patient, indicating clonality and suggesting acquisition early during tumor evolution.

The remaining mutations were identified in cervical neuroendocrine carcinoma, head and neck squamous cell carcinoma, metastatic bladder carcinoma, and gastric neuroendocrine carcinoma. Collectively, these findings demonstrate that rare AF1 mutations occur across multiple epithelial tumor types.

### 3.2. AF1 Mutations Occur Within a Proline-Rich Phosphoregulatory Segment

**Figure 1:**
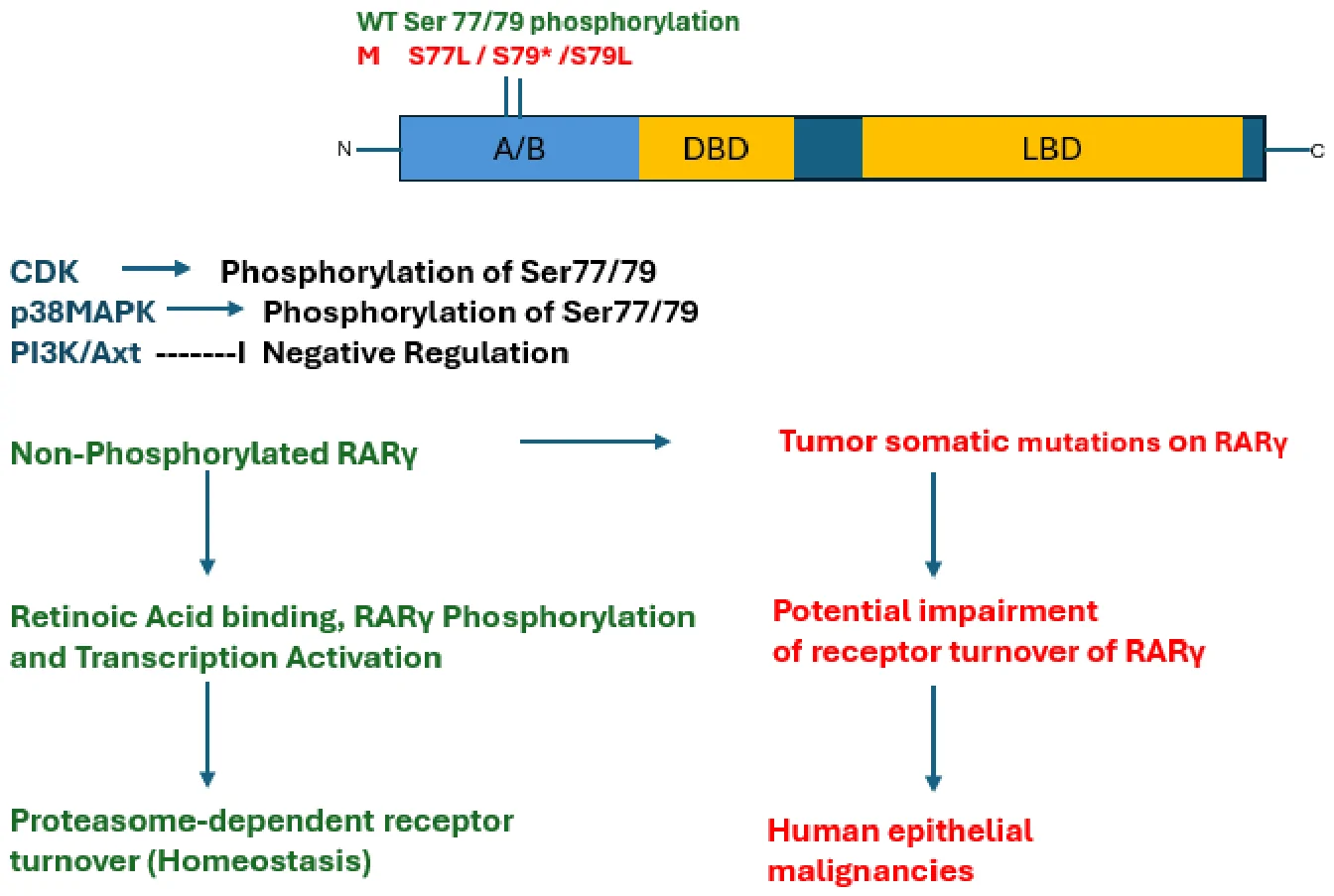
Mechanistic model linking the experimentally validated RARγ phosphoregulatory module to naturally occurring cancer-associated mutations. Ligand binding induces sequential phosphorylation of Ser77 and Ser79 (Ser66 and Ser68 in RARγ2), promoting receptor activation, ubiquitination, and proteasome-dependent receptor turnover as established by previous mechanistic studies. The present study identifies naturally occurring somatic mutations affecting these experimentally validated phosphoregulatory residues in human epithelial malignancies, supporting the hypothesis that disruption of this regulatory module may impair receptor turnover and alter RARγ signaling.

The experimentally validated phosphoregulatory residues Ser77 and Ser79 are embedded within a short proline-rich segment of the AF1 domain (Figure 1). In addition to mutations directly affecting the phosphoregulatory serine, sequence variation has been reported at neighboring residues within this region, including several proline residues flanking the phosphorylation sites.

Because proline residues play important roles in peptide backbone organization and local conformational properties, the sequence context surrounding Ser77 and Ser79 may contribute to the regulatory behavior of the AF1 domain. Although the consequences of neighboring variants remain unknown, the localization of rare cancer-associated mutations within this proline-rich segment further emphasizes the functional importance of this AF1 region (Figure 1).

## 4. Discussion

Somatic alterations involving the experimentally validated phosphoregulatory residues Ser77 and Ser79 were detected in multiple epithelial malignancies, including cervical neuroendocrine carcinoma, head and neck squamous cell carcinoma, lung adenocarcinoma, bladder carcinoma, and gastric neuroendocrine carcinoma. These observations extend the spectrum of cancer-associated RARG alterations beyond receptor overexpression and leukemia-associated gene rearrangements and identify the AF1 domain as a previously underappreciated target of mutations in human cancer.

A notable feature of these findings is the relationship between the identified mutations and previous mechanistic studies of RARγ regulation [6,18]. More than two decades ago, biochemical and molecular studies demonstrated that phosphorylation of Ser66 and Ser68 in RARγ2, corresponding to Ser77 and Ser79 in canonical RARγ1, is required for ligand-dependent receptor activation, ubiquitination, and proteasome-mediated degradation. These residues were shown to integrate TFIIH/CDK7-, p38MAPK-, and PI3K/Akt-dependent regulatory pathways controlling receptor turnover and transcriptional activity [6,7,8,9]. The present study demonstrates that human tumors harbor naturally occurring mutations affecting the same experimentally validated regulatory segment, thereby extending earlier observations from receptor biology to human malignancy.

RARγ has increasingly emerged as a cancer-associated gene. Studies of solid tumors have implicated elevated RARγ expression in tumor progression, cancer stem-cell maintenance, metastasis, and adverse clinical outcome, while studies of acute myeloid leukemia have demonstrated recurrent oncogenic RARG rearrangements that define a distinct disease subtype with poor prognosis and resistance to retinoid-based therapy [1,2,3,4,5]. Most of these investigations have focused on receptor abundance, signaling pathways, or fusion proteins involving the DNA-binding and ligand-binding domains. In contrast, little attention has been directed toward the AF1 domain. The present findings therefore expand the landscape of cancer-associated RARγ alterations by introducing AF1-domain mutations as an additional mechanism through which this receptor may be altered in human tumors.

An additional observation is that the phosphoregulatory residues are located within a proline-rich AF1 segment. Besides mutations directly affecting Ser77 and Ser79, neighboring sequence variants have been reported within the same region. Because proline residues contribute to peptide backbone organization and local conformational flexibility, the sequence context surrounding the phosphoregulatory residues may participate in AF1 regulatory functions [19]. Although the biological significance of neighboring variants remains unknown, the structural organization of this short segment highlights an area that may warrant further biochemical and structural investigation.

Among the identified cancer-associated mutations, the p.S79* truncating alteration is particularly noteworthy because it was detected in five spatially distinct regions of a single EGFR-mutant lung adenocarcinoma. The presence of the mutation across multiple tumor regions is consistent with clonal acquisition during tumor evolution and suggests that disruption of the AF1 phosphoregulatory region can occur early in at least some tumors. Nevertheless, the rarity of all identified AF1 mutations indicates that they are likely to define uncommon molecular events rather than a frequent mechanism of oncogenesis.

Several limitations should be acknowledged. The number of identified cases is small, consistent with the generally low frequency of RARG mutations in human cancer. In addition, no direct functional studies have yet examined the effects of the cancer-associated AF1 mutations identified here. Future experimental studies will be required to determine how these mutations influence phosphorylation, receptor turnover, transcriptional activity, and cellular responses to retinoid signaling.

The functional significance of Ser79 is not inferred solely from computational analyses. Prior biochemical studies demonstrated that Ser79 phosphorylation regulates RARγ transactivation, ubiquitination, and degradation [6]. The present work identifies naturally occurring cancer-associated mutations affecting this experimentally validated phosphoregulatory region.

## 5. Conclusions

In conclusion, the present study identifies rare cancer-associated mutations affecting the AF1 phosphoregulatory domain of RARγ, including residues previously demonstrated to regulate receptor activation and proteasome-mediated degradation. By linking contemporary cancer genomics with a regulatory module characterized experimentally more than twenty-five years ago, these findings extend the known spectrum of cancer-associated RARG alterations and, by linking contemporary cancer-genomics observations with a previously characterized phosphoregulatory module of RARγ, the present study identifies rare cancer-associated variants occurring within the AF1 domain and highlights this region as a potential subject for future investigations.

## Figures and Tables

**Table 2:** COSMIC mutations at Ser77 (RARγ1), corresponding to Ser66 in RARγ2.

Position (AA)	CDS Mutation	AA Change	COSMIC ID	Count	Type
77	c.230C>T	p.S77L	COSM8337174	2	Substitution—Missense
78	c.230_231insCTTGCA	p.S77_P78insLQ	COSM10562079	1	In-frame insertion

The p.S77L missense variant was identified in two independent patient tumors, whereas the p.S77_P78insLQ variant was reported as an in-frame insertion spanning Ser77 and Pro78; however, its somatic status was not confirmed in the available COSMIC annotation. This event is an in-frame insertion spanning the phosphoregulatory residue and the adjacent Pro78. The insertion should be interpreted cautiously because its somatic status was not confirmed in the available COSMIC annotation.

**Table 3:** COSMIC mutations at Ser79 (RARγ1), corresponding to Ser68 in RARγ2.

Position (AA)	CDS Mutation	AA Change	COSMIC ID	Count	Type
79	c.236C>A	p.S79*	COSM7410973	1 (five spatially distinct tumor regions)	Substitution—Nonsense (stop gain)
79	c.236C>T	p.S79L	COSM11084365	1	Substitution—Missense
79	c.237G>A	p.S79=	COSM8801005	1	Substitution—Coding silent

At Ser79, COSMIC records a truncating p.S79* event across five spatially distinct regions from a single lung adenocarcinoma, one p.S79L missense variant in bladder carcinoma, and one synonymous p.S79= variant.

**Table 4:** Clinical characteristics of reported cases harboring AF1-region variants.

Mutation	COSMIC ID	Tumor Type *	No. of Patients	Stage *	Histology *	Key Details	Source
S79* (RARγ1) = S68* (RARγ2)	COSM7410973	Lung adenocarcinoma	1 patient (5 multi-region samples)	Stage IIA	Adenocarcinoma	EGFR+, Asian female, non-smoker, age 72, clonal mutation	Nahar et al., 2018 (PMID 29335443) [13]
S79L (RARγ1) = S68L (RARγ2)	COSM11084365	Urinary tract—Bladder (transitional cell carcinoma)	1	Metastatic (by cohort design)	Transitional cell carcinoma	Male, age 74, whole-genome duplication, MSI-stable	Priestley et al., 2019—Pan-cancer metastatic WGS (PMID 31645765) [14]
S77_P78insLQ (RARγ1)—in-frame insertion	COSM10562079	Stomach—Gastric neuroendocrine carcinoma	1	Not recorded	Stomach (carcinoid-endocrine tumor)	Heterozygous; not yet confirmed somatic	Griger et al., 2023—Gastric NEC evolution (PMID 37352862) [15]
S77L (RARγ1) = S66L (RARγ2)	COSM8337174	Cervical carcinoma (neuroendocrine)	1	Stage IIIC1	Poorly differentiated, admixed neuroendocrine carcinoma	Female, age 36, HPV+, MSI-stable	iPTMnet. Report for P13631 [16]
S77L (RARγ1) = S66L (RARγ2)	COSM8337174	HNSCC/Head-neck (squamous cell)	1	Not recorded	Squamous cell carcinoma	Male, age 74, TCGA-HNSC cohort	TCGA via COSMIC/cBioPortal [17]

* All clinical variables shown in Table 4, including tumor type, histology, patient numbers, and stage, were obtained from COSMIC annotations associated with the corresponding mutation records. The specific staging system and edition were not reported in the publicly available COSMIC annotations for these cases and therefore could not be independently verified.

## Data Availability

All data analyzed during this study were obtained from publicly available resources, including COSMIC, iPTMnet, and cited primary publications. No new datasets were generated.

## Abbreviations

The following abbreviations are used in this manuscript:

AF1	Activation Function-1
WGS	Whole-Genome Sequencing
TFIIH	Transcription Factor IIH
AML	Acute Myeloid Leukemia
TCGA	The Cancer Genome Atlas
ATRA	All-Trans Retinoic Acid
COSMIC	Catalogue Of Somatic Mutations In Cancer
DBD	DNA-Binding Domain
EGFR	Epidermal Growth Factor Receptor
HNSCC	Head and Neck Squamous Cell Carcinoma
HPV	Human Papillomavirus
LBD	Ligand-Binding Domain
MSI	Microsatellite Instability
PTM	Post-Translational Modification
RAR	Retinoic Acid Receptor
RARγ	Retinoic Acid Receptor Gamma
RARG	Retinoic Acid Receptor Gamma Gene
RXR	Retinoid X Receptor
SCC	Squamous Cell Carcinoma
